# VIRTUAL COMPREHENSIVE GERIATRIC ASSESSMENT FOR VETERANS: AN INTERDISCIPLINARY QUALITY IMPROVEMENT PILOT PROJECT

**DOI:** 10.1093/geroni/igad104.2316

**Published:** 2023-12-21

**Authors:** Maria Cervantes, Janette Dunlap, Robynn Scott, Teresa Howell, Amy Neal, Heather Bieber, Jacob Tillmann

**Affiliations:** Veterans Health Administration, Saint Johns, Florida, United States; The North Florida/South Georgia Veterans Health System, Jacksonville, Florida, United States; The North Florida/South Georgia Veterans Health System, Jacksonville, Florida, United States; North Florida/South Georgia Veterans Health System, Gainesville, Florida, United States; The North Florida/South Georgia Veterans Health System, Jacksonville, Florida, United States; Department of Veterans Affairs, Lake City, Florida, United States; Department of Veterans Affairs, Gainesville, Florida, United States

## Abstract

The Veterans Millennium Health Care and Benefits Act requires comprehensive geriatric assessments (CGA) to be provided to all older Veterans as part of the Veterans Medical Benefits Package. Access to a CGA may be difficult for Veterans with limited ability to travel. Currently, in the North Florida/South Georgia (NFSG) Veterans Health System, approximately 11,500 older Veterans are recognized to be at high risk for hospitalization or placement in a long-term care facility, yet only 0.09% of them have received a CGA. NFSG Geriatrics and Extended Care (GEC) developed a standardized virtual CGA process to increase access to geriatric specialty care utilizing VA Video Connect (VVC). The project design and implementation included: the development of a standard operating procedure using evidence-based screening tools, templates, clinic grids and flowcharts, communication channels, and the development of a dashboard for data collection. In addition, the VHA National CGA Template was piloted. This novel Tele-Geriatrics virtual interdisciplinary project allows older Veterans and/or caregivers to remain at their homes for all telehealth/virtual sessions completed by the CGA interdisciplinary team, including pharmacy, nursing, social work, occupational therapy, and geriatric providers. The project demonstrates increased access to comprehensive assessments, consequently closing the gap between current practice and the Veterans Millennium Health Care and Benefits Act requirements. The pilot also decreased the health disparities of rural Veterans. Furthermore, this project helped to increase Veteran knowledge of geriatric syndromes, VA resources, participation in telehealth, and access to durable medical equipment (DME) to help Veterans age in place.

